# ‘I love you’: the first phrase detected from dreams

**DOI:** 10.5935/1984-0063.20220035

**Published:** 2022

**Authors:** Michael Raduga

**Affiliations:** REMspace, Phase Research Center - Novorossiysk - Krasnodar Krai - Russia.

**Keywords:** Dreams, Lucid Dreaming, Language, Sleep, REM, Vocalization, Consciousness

## Abstract

**Objective:**

Many people have dreams nightly and some maintain consciousness during dreams. Such dreams are referred to as lucid dreams (LD). During dreams, our speech correlates with facial muscle activity, which is hard to decode, but LD could solve this problem. The primary hypothesis of this study was that the facial muscles electric activity during LD corresponds to specific sounds. Understanding this connection could help decode dream speech in the future.

**Material and Methods:**

Under laboratory conditions, four LD practitioners were asked to say “*I love you*”, a phrase with a distinctive electromyographic (EMG) signature. They did this before falling asleep and then again after becoming conscious during a dream. Their facial and neck EMG was recorded in four areas.

**Results:**

All four volunteers accomplished the goal at least once. The patterns associated with the “*I love you*” phrase were observed in most cases, both during wakefulness and LD. Specifically, the “*I*” triggered distinctive phasic activity in the *submentalis* area most of the time, while “*you*” did the same in the *orbicularis oris*.

**Discussion:**

This study highlights the possibility of detecting only specific and highly EMG distinctive phrases from dreams because vocalization also involves a tong and vocal apparatus. The most interesting consequence of the present results is that they indicate the possibility of creating an artificial EMG language that could be instantly decoded in reality and used during LD.

## INTRODUCTION

Dreams are emotions and perceptions experienced during sleep. A person’s dream recall frequency (DRF) usually rises from adolescence to early adulthood before decreasing by the age of 50-60 years^1^. DRF depends not only on age^2-6^ but on gender^1,7,8^, pathologies^6,9^, individual brain structure^10,11^, stress^12^, sleep quality^13^, ultradian NREM-REM sleep cycle and the circadian modulation of REM sleep^14^, cultural differences^15^, and other factors. For a long time, it was believed that dreams happen only during REM sleep. Though this is still true of vivid dreams, we now know dreams can be recalled after other sleep stages^16-20^, even after slow-wave sleep^21-23^.

Early research revealed that our eyes follow dream scenes^24,25^, as the eye muscles are not paralyzed during REM sleep^26,27^. The most significant achievement in this research direction was the creation of a neural decoding approach that uses machine learning to predict the contents of visual imagery during sleep onset^28^. In the future, functional magnetic resonance imaging can be used to enhance similar technologies^29^. A correlation between talking during dreams and chin electromyography (EMG) was established long ago^30,31^. Thus, EMG could help us not only to ‘hear’ our dreaming voice but also to decode our movements in dreams^32^.

The main problem with most of the abovementioned studies is the absence of a direct connection between dreamers and researchers. As a result, it is hard to understand what exactly a person does in a dream. If we could determine this, it would help to correlate recorded data with actual actions or perceptions in dreams. This problem could be solved by lucid dreams (LD) because not only can people see LD, but they can also control them^33,34^. LD usually happen during REM sleep, though they can occur during non-REM sleep in rare cases^35-37^, and can be triggered by acetylcholine^38,39^.

The term ‘lucid dreaming’ was first used by Frederik Van Eeden, in 1913^40^, though the phenomenon was not confirmed by science until 1975. LD were confirmed when pre -agreed eye movements (PAEM) were observed during REM sleep^41^. Recent studies show that LD have several practical applications, such as preventing nightmares^42,43^, lowering chronic pain^44^, problemsolving^45,46^, controlling computers while asleep^47^, and exercising motor skills^48^.

A meta-analysis of 50 studies shows that 55% of people have experienced at least one LD^49^. Several other phenomena share LD’ primary features (e.g., consciousness during REM sleep), including sleep paralysis^50,51^, false awakenings^52^, and out-of-body experiences^53-55^. These states are grouped under the term *phase state* (PS)^55,56^ or *dissociated REM state*^57^. A survey revealed that 88% of people have experienced at least one phase state; 43% of people experience one or more of them often^58^.

LD have been successfully used since the late 1970s to establish connections between the dream world and wakefulness. For example, Hearne (1978)^41^ detected such a connection based on PAEM. Furthermore, LaBerge et al. (1981)^33^ found that Morse signals induced in the arm muscles could be transferred from LD into reality. Later, it became possible to communicate with people during LD in real time using much broader approaches involving breathing and facial muscle expressions^59-61^.

If facial EMG is connected to dream speech^30,31^, we might be able to use LD to identify specific sounds, words, or even phrases from dreams. As far as we know, no scientific attempts have been made to do this, though some studies present promising preliminary data. For example, a process has been developed for digitally voicing silent speech, which could work similarly for dream speech. Gaddy and Klein (2020)^62^ showed that the EMG patterns of silent speech are correlated with the patterns of vocalized speech - this knowledge could be used to improve vocalized speech. Such technologies could be useful for people who have had their larynx removed^63^ and for creating silent speech-to-text systems^64^.

### Hypotheses

The primary hypothesis of this study is that speech vocalized in an LD presents the same EMG patterns as speech vocalized during wakefulness. Confirmation of this hypothesis could provide a method for decoding LD speech, as well as speech from unconscious dreams. The results of this research could also improve our understanding of dreams, LD, REM sleep, consciousness, and vocalization. It could also open new opportunities for ‘seeing’ the dream world.

## MATERIAL AND METHODS

### Resources and participants

The present research was accomplished under laboratory conditions using facial and neck EMG sensors. Highly experienced LD practitioners were invited to volunteer in the study. LD practitioners are people who are trained to induce LDs by their will. They were chosen based on their ratings in the Project Elijah online platform, where LD practitioners continuously accomplish different LD experiments. We contacted and invited to participate in the present study those practitioners who had accomplished the most experiments and lived close to the laboratory. Ethical committee approval was obtained from the Phase Research Center’s institutional review board (approval’s ID: PRC-2021-6-11-02).

All volunteers confirmed that they were at least 18 years old and presented no psychological or physiological issues that could be affected by LD or the study protocol. Written informed consent was obligatory for all subjects before the research began. Volunteers’ primary motivation for participating was the opportunity to explore their LD practice. No financial reward was given, though all travel and accommodation expenses were covered.

### Apparatus and LD detection

**Figure 1:**
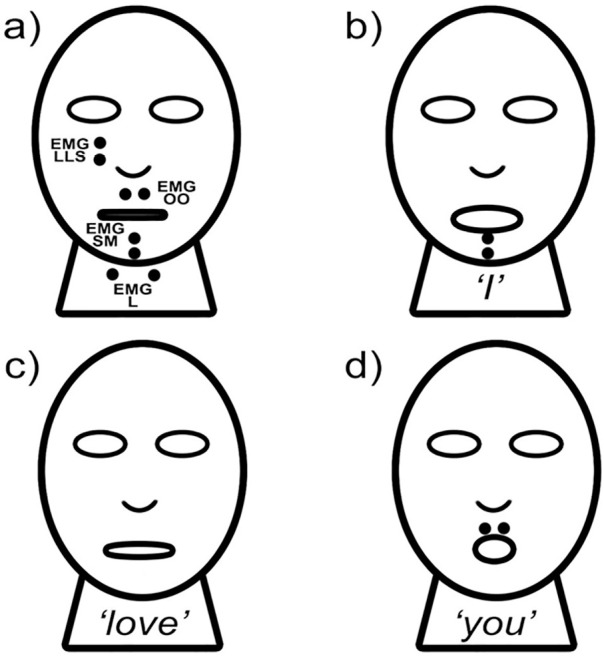
A EMG sensors placement: EMG LLS = *Levator labii superioris* area; EMG OO = *Orbicularis oris* area; EMG SM = *Submentalis* area; EMG L = *Laryngeal* area; **B.** Distinctive EMG SM activation during “*I*” vocalization; **C.** No distinctive EMG activation during “*love*” vocalization; **D.** Distinctive EMG OO activation during “*you*” vocalization.

‘Encephalan-EEGR-19/26’ was used to accomplish the research goals by tracking EMG and detecting LD occurrences. The configuration contained four EMG channels (50Hz notch filter; 16-70Hz band-pass filter) (Figure 1a). EMG #1 was located in the *levator labii superioris* area (EMG LLS). EMG #2 was located in the *orbicularis oris* area, under the upper lip (EMG OO). EMG #3 was located in the *submentalis* area (EMG SM). EMG #4 was located in the *laryngeal* area in front of the vocal apparatus (EMG L). These EMG locations were used due to their efficiency in silent speech^62^.

### Experimental task

**Figure 2:**
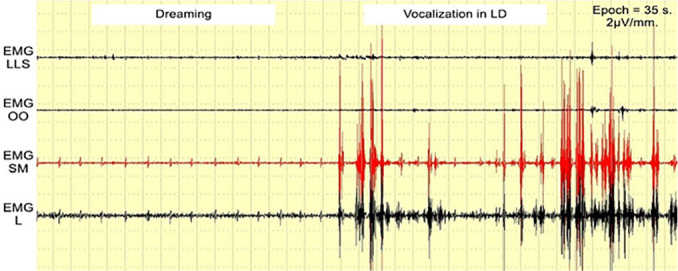
A preliminary test with successful loud vocalization in LD.

Abbreviation: EMG LLS = *Levator labii superioris* area; EMG OO = *Orbicularis oris* area; EMG SM = *Submentalis* area; EMG L = *Laryngeal* area.

**Figure 3:**
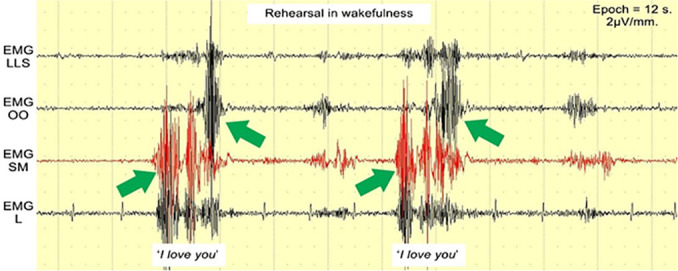
The ‘*I love you*’ pattern in wakefulness.

Abbreviations: EMG LLS = *Levator labii superioris* area; EMG OO = *Orbicularis oris* area; EMG SM = *Submentalis* area; EMG L = *Laryngeal* area.

Preliminary tests, including successful vocalization during an LD (Figure 2), indicated that any sounds made during LD should present distinctive EMG patterns if they are to be detected clearly. The phrase *“I love you”* was used for this preliminary test. While *“love”* could change its pattern from time to time (Figure 1c), *“I”* could mostly trigger EMG SM (Figure 1b), and “*you*” could do the same with EMG OO (Figure 1d), thereby creating a stable and distinctive EMG pattern in wakefulness (Figure 3), which, in theory, could be seen from LD vocalization as well. EMG LLS and EMG L were used to gather more data from the study, even though their correlation with *“I love you”* pronunciation was week.

LD practitioners received an instructed to perform the following procedure after assembling EMG sensors: A) clearly pronounce *“I love you’* a few times and perform pre -agreed chin movements (PACM); B) fall asleep and induce an LD by any convenient technique like creating a strong intention upon falling asleep to become conscious in an upcoming dream; C) if B is successful, loudly pronounce “*I love you”* a few times during the LD and perform PACM; D) if C is successful, try to enter another LD and repeat step C. Volunteers were able to focus on the study goals across one to three nights in a laboratory. No limitations were imposed in terms of LD quantity.

The volunteers were also allowed to use LD maintaining and stabilizing techniques to prolong LD and increase their quality.

### PACM as an LD detection method

PACM require EMG sensors to be placed only in the *submentalis* area, which was already included in the configuration of the current experiment. REM sleep atonia and phasic bursts of PACM can be detected during LD using the PACM method^65^. As REM sleep and consciousness are the main characteristics of LD, the PACM method is considered the main LD detection method in the present study. Volunteers were asked to deploy three consecutive and wide chin movements while in an LD to manifest consciousness during sleep atonia.

## RESULTS

Four volunteers participated in the study, sleeping from one to three nights in the laboratory with EMG sensors attached (24-40 years old; females N=2). All four volunteers were able to experience LD and achieve the goal at least once. One of the volunteers was able to achieve the goal twice. All the reports showed distinctive phasic EMG bursts during vocalization in LD and PACMs.

**Figure 4:**
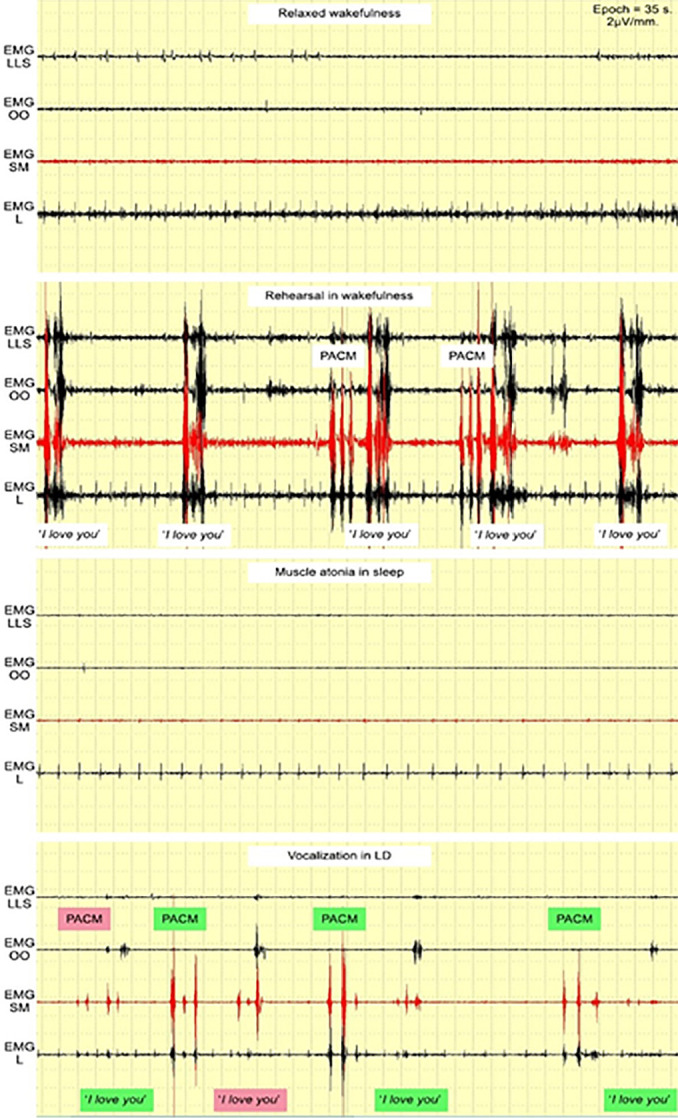
First LD of volunteer #1.

Abbreviations: EMG LLS = *Levator labii superioris* area; EMG OO = *Orbicularis oris* area; EMG SM = *Submentalis* area; EMG L = *Laryngeal* area.

**Figure 5:**
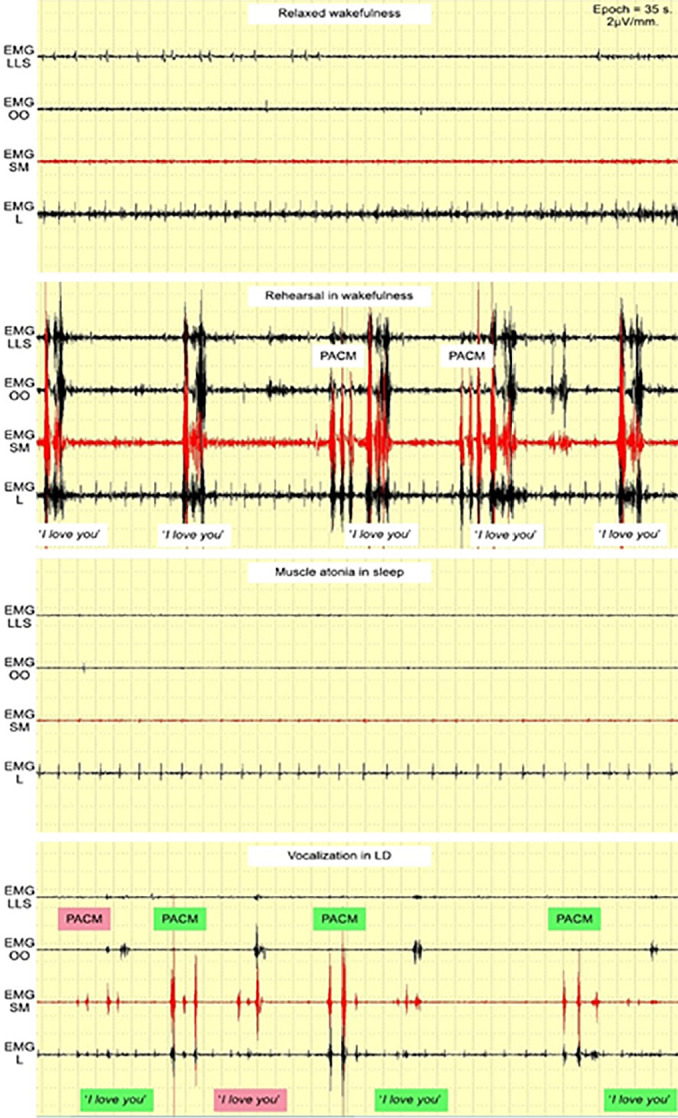
Second LD of volunteer #1.

Abbreviations: EMG LLS = *Levator labii superioris* area; EMG OO = *Orbicularis oris* area; EMG SM = *Submentalis* area; EMG L = *Laryngeal* area; PACM = Pre-agreed chin movements.

Volunteer #1 reported their first LD on the first night and performed the PACM/vocalization cycle four times. The *“I love you”* EMG pattern fully coincided three times and 50% one time. In one unsuccessful vocalization attempt, “*you*” was stronger for EMG SM than EMG OO, though both were powerful (Figure 4). No LD occurred on the second night for this volunteer. The second LD occurred on the third night, and the volunteer performed the PACM/vocalization cycle four times. The *“I love you”* EMG pattern fully coincided two times and 50% the other two times. In both unsuccessful vocalization attempts, the EMG SM burst of “*I*” was not distinctive enough (Figure 5).

**Figure 6:**
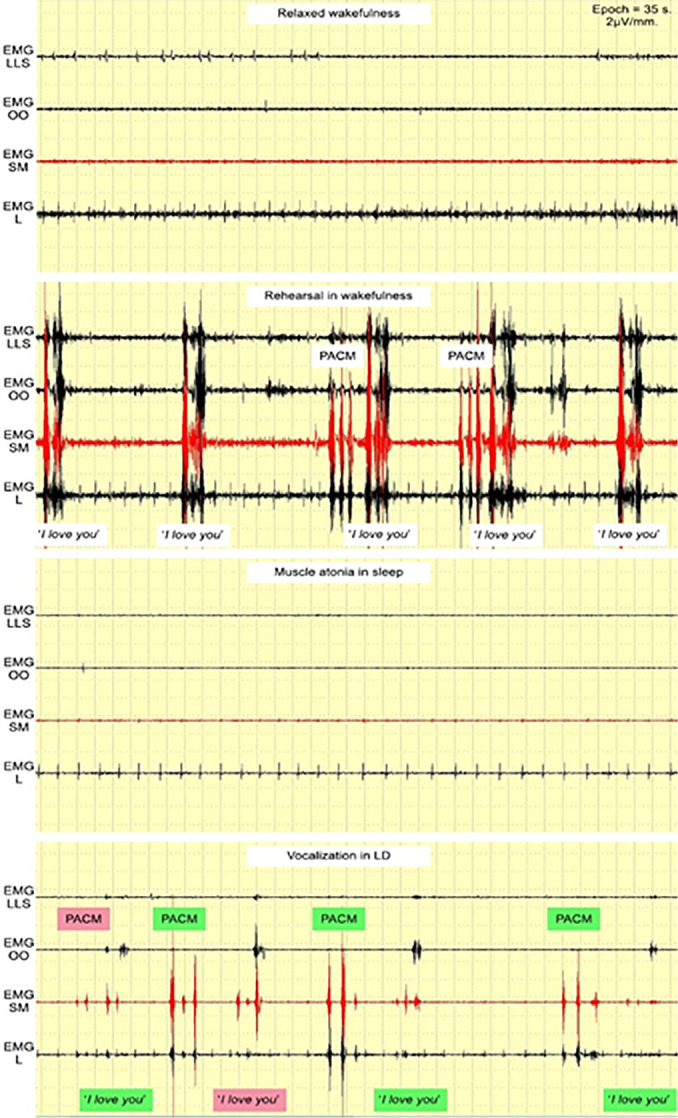
LD of volunteer #2.

Abbreviations: EMG LLS = *Levator labii superioris* area; EMG OO = *Orbicularis oris* area; EMG SM = *Submentalis* area; EMG L = *Laryngeal* area; PACM = Pre-agreed chin movements.

Volunteer #2 reported one LD on the first night and performed PACM/vocalization cycle three times. The “*I love you”* EMG pattern fully coincided two times and 50% the other time. In one unsuccessful vocalization attempt, the EMG SM burst of *“I”* was not distinctive enough (Figure 6). The volunteer reported very loud vocalization (close to shouting) in LD.

**Figure 7:**
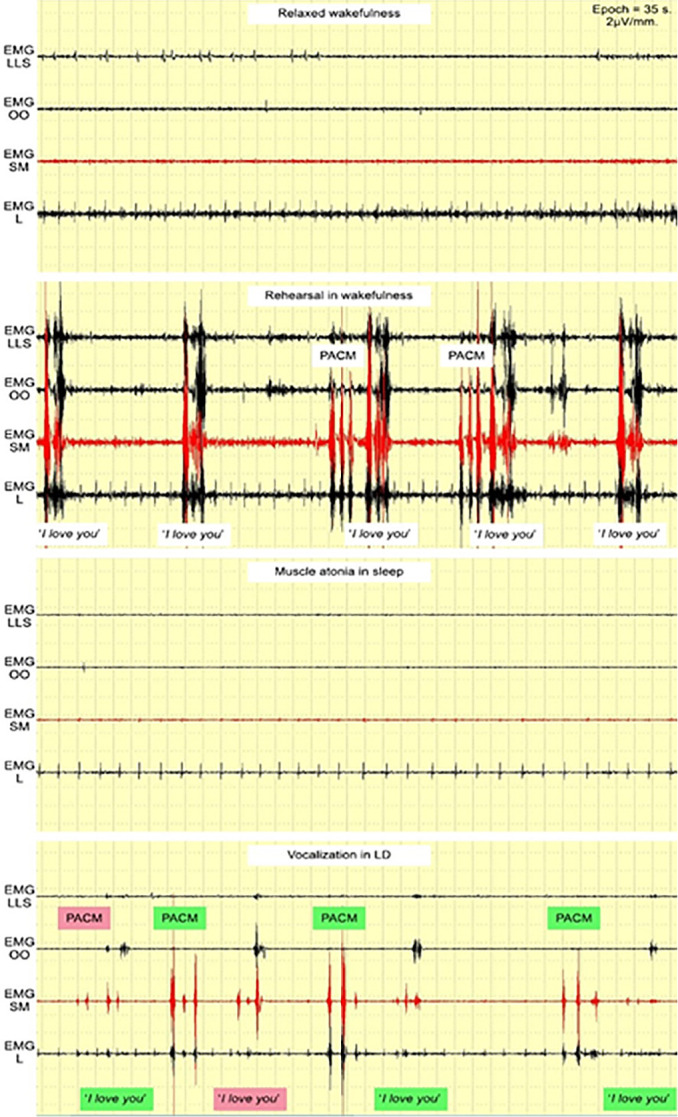
LD of volunteer #3.

Abbreviations: EMG LLS = *Levator labii superioris* area; EMG OO = *Orbicularis oris* area; EMG SM = *Submentalis* area; EMG L = *Laryngeal* area; PACM = Pre-agreed chin movements.

Volunteer #3 reported one LD on the second night and performed PACMs followed by two vocalizations three times each. During the first vocalization, only PACMs were distinctive. During the second vocalization, which happened 30 seconds after the first, the “*I love you”* EMG pattern fully coincided two times and 50% one time. In one unsuccessful vocalization attempt, the EMG SM burst of *“I”* was not distinctive enough (Figure 7).

**Figure 8:**
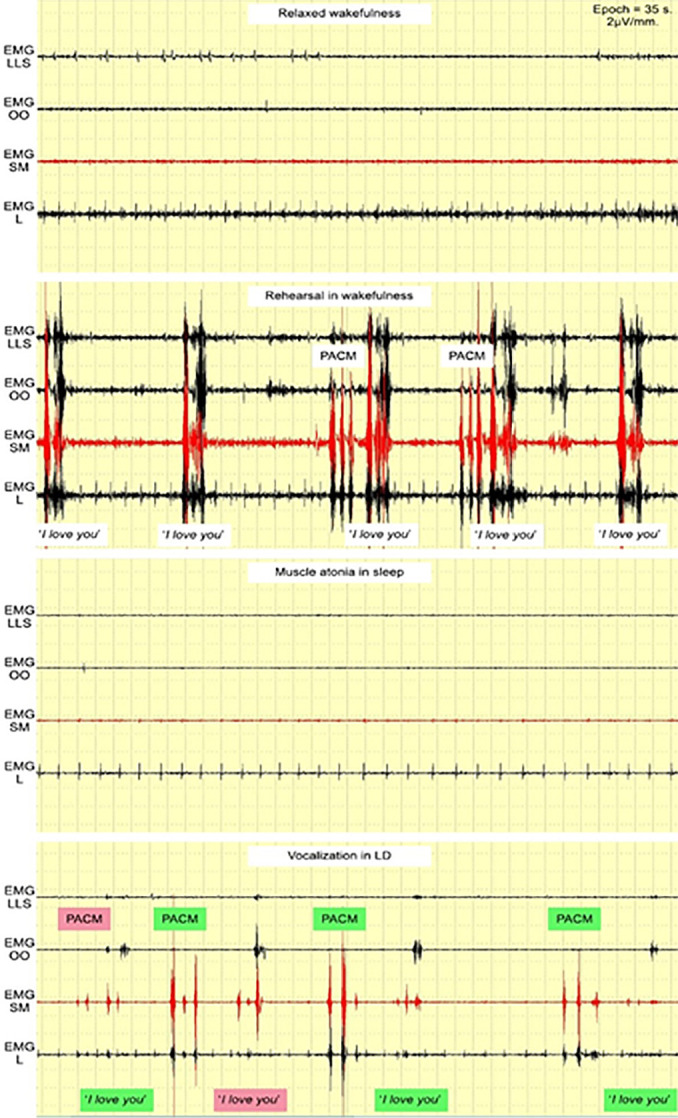
LD of volunteer #4.

Abbreviations: EMG LLS = *Levator labii superioris* area; EMG OO = *Orbicularis oris* area; EMG SM = *Submentalis* area; EMG L = *Laryngeal* area; PACM = Pre-agreed chin movements.

Volunteer #4 reported one LD on the first night, during which they performed two PACM/vocalization cycles right before awakening. The EMG data analysis indicated that only the first PACM were hardly distinctive. Both vocalization patterns of *“I love you”* coincided at 50%, as EMG OO activity was missing (Figure 8). The volunteer expressed that their vocalization level ‘could be higher.’ During the second night, the volunteer could not enter an LD.

## DISCUSSION

Humanity has always been fascinated by dreams, hoping to see them in reality. Technological advances have given us tools to glimpse into dreams, but we still have not had any significant success. Due to EMG-related correlations between the physical facial muscles and dream speech, we might at least be able to ‘hear’ what we talk about while we are asleep. LD could help with this goal, as speech can be controlled during LD. We hypothesized that an EMG distinctive phrase could present the same pattern in wakefulness as in an LD.

### Hypotheses confirmation

The results show relatively stable EMG correlations when pronouncing the goal phrase in wakefulness and in an LD when the phrase was vocalized loudly and with concentration in both cases. “*I love you”* is the first phrase that we detected from dreams with certainty - we knew it was said in dreams and we detected its EMG pattern at the same time. Thus, it is reasonable to conclude that LD can be used to decode dream speech. From the perspective of previous studies confirming the correlation between facial EMG activity dream speech^30-32^, the present results are not unexpected.

All narratively reported successful LD with vocalization were found in the EMG data, and all cases were highly distinctive from normal EMG sleep patterns. Rare phasic spikes occurred during most low tonic activity, which most likely represented sleep atonia. Apparent phasic activity was detected when vocalizations were reported.

We certainly cannot effectively detect and decode most dream speech using the EMG settings of the current study. Vocalization requires the tongue and vocal apparatus in addition to facial muscles - therefore, examining facial muscles via facial EMG alone is not enough to decode all sounds. However, future technological advances could make this kind of decoding possible.

Nevertheless, the results of the present study indicate the current possibility of detecting EMG patterns for some specific sounds. However, different sounds can have the same facial EMG patterns. Therefore, more advanced and complicated settings, including machine learning, are needed to differentiate all the sounds. The current study only confirms that this method could be developed in the future. For now, we know that speech in dreams indeed coincides with the EMG patterns of vocalizations in reality.

EMG L, located near the vocal apparatus, did not consistently show any specific activity that was distinctive from the activity of other EMG sensors. In most cases, EMG L activity in wakefulness and during LD was closely related to EMG SM activity - these two EMGs were located close to one another.

### New research opportunities

The current study presents impressive perspectives of LD practitioners. However, new studies with more advanced technologies are needed to effectively decode English (or any other language) from ordinary dreams. If LD are studied (as opposed to regular dreams), the findings of the present study could already be applicable. This is because people can control their vocalization and deploy specific EMG patterns during LD. Therefore, some distinctive EMG pre-agreed phrases can be learned and detected using just a few sensors.

This branch of research could become even more interesting if an EMG language is developed for LD practitioners. In theory, this language could use only a few distinct EMG sounds, which all could be distinctively vocalized, efficiently read by sensors, and automatically decoded. This artificial language could make it possible to ‘hear’ everything pronounced in LD. Eventually, LD practitioners could be able to communicate with each other over the Internet while asleep^55,56^. In this case, an artificial EMG language could be pivotal - the results of the current study confirm its possibility for general use.

Vocalization in LD requires not only conscious actions but also EMG traces on the face, *submentalis*, and throat - these last two also can be used to detect sleep atonia adherent to REM sleep^66^, which in turn is the main physiological sleep stage associated with LD^67^. Pre-agreed vocalization (PAV) during LD could be used together with PAEM and PACM detection methods. For example, after LD vocalization, facial EMG patterns, which are very unlikely to happen spontaneously during ordinary dreams, were detected. These patterns could serve as a clear sign of consciousness alongside with PAEM or PACM.

### Vocalization in other phase states

As LD naturally relate to phenomena like sleep paralysis (SP), out-of-body experiences, and false awakenings^58^, the results of the current study could apply to all these states. This is especially important regarding SP, which is frequent among the general population^68,69^ and presents a severe problem for narcoleptics^70^. The outcomes of the present study indicate that a vocal/EMG communication tool could be developed for people suffering from SP. In theory, this could enhance SP studies and reduce the fear experienced during SP through bidirectional communication with reality.

### Limitations

The most controversial issue presented in this study is the implementation of PACM to detect LD, so no electroencephalography (EEG) or electrooculography (EOG) were recorded. Thus standard sleep staging could not be done. In this sense, our results should be viewed as preliminary. This was the first study to use PACM approach, excluding PACM testing itself^65^. In most previous LD studies, PAEM were used as the standard verification method (for a review see MotaRolim, 2020^71^). This method requires EEG and EMG to detect sleep stages and EOG to detect consciousness. Therefore, an alternative PACM protocol was used, because this method significantly reduced the number of cords and sensors placed on the volunteers’ heads and faces during this particular study. Muscle atonia reflects REM sleep and pre-agreed phasic signals through EMG SM represent consciousness. Thus, both primary attributes of LD are confirmed alongside the narrative reports of the volunteers.

Subjects were asked to pronounce the goal phrase very loudly and distinctively to ensure the phrase could be detected during the LD. They also practiced for a couple of minutes before falling asleep. However, even after these instructions, the phrase was not always pronounced loudly, and the resultant EMG patterns were very week (Figures 5 and 8), meaning that quiet speech during LD and ordinary dreams could not be detected.

So, in the current study, vocalization in dreams was detected only if the phrase was vocalized loudly and distinctively. Vocalizations that were close to shouting were especially effective (Figure 6). In future research and practical applications, LD practitioners should rehearse the phrases even more and vocalize them very distinctively while dreaming, feeling the muscle tension related to each specific sound. This strategy will yield more distinctive results than the present study, with fewer mistakes.

In the present study, the most prominent problem with vocalization during LD was associated with EMG SM phasic activity. This vocalization problem occurred more often than with EMG OO.

## CONCLUSION

The study highlights a new opportunity to explore dreams with the help of LD. Though we still cannot see dreams from reality, it seems possible to partially ‘hear’ what we say in dreams through facial EMG patterns. However, we cannot detect any sounds or words using this new tool because the vocalization process also involves complicated functions of the tongue and vocal apparatus, which cannot currently be decoded from dreams. Regardless, this new LD tool could be useful for detecting highly distinctive EMG sounds, words, and phrases. In turn, this could be useful for understanding dreams, as well as helping people who suffer from sleep paralysis.

The results of the present study could present new opportunities for future studies. First, we should clarify exactly why it is possible to read EMG traces of vocalizations in dreams. Second, it would be helpful to study minute details related to the topic (e.g., which sounds can be decoded, the proper way to vocalize sounds, whether an EMG dream vocabulary can be created). Very intriguing opportunities could arise if we find or create a way to use EMG to decode all words in dreams. This would provide LD practitioners with a way to communicate with each other while sleeping. Perhaps an artificial EMG language should be designed to achieve this goal. Also, it could be explored whether we can use LD to hone our speech, given that silent speech improves pronunciation.

Exploring new opportunities via vocalizations during LD could provide more chances for decoding dreams. It could also improve our knowledge about the sleep process, REM sleep, muscle atonia, vocal apparatus, and many other topics. Therefore, new hypotheses based on the current study could help science and culture to reach new horizons.
